# Nephrectomy with Autotransplantation—A Key Treasure

**DOI:** 10.3390/jcm13061641

**Published:** 2024-03-13

**Authors:** Sofia Mesquita, Miguel Marques-Monteiro, Mariana Madanelo, Maria Alexandra Rocha, Nuno Vinagre, Avelino Fraga, Vítor Cavadas, Rui Machado, Miguel Silva-Ramos

**Affiliations:** 1 Department of Urology, Unidade Local de Saúde de Santo António, 4099-001 Porto, Portugal; mmonteiro.iam@gmail.com (M.M.-M.); u13015@chporto.min-saude.pt (M.M.); malexandrarocha.urologia@chporto.min-saude.pt (M.A.R.); u14536@chporto.min-saude.pt (N.V.); avelinofraga.urologia@chporto.min-saude.pt (A.F.); vcavadas@gmail.com (V.C.); 2 Instituto de Ciências Biomédicas Abel Salazar (ICBAS), 4050-313 Porto, Portugal; rmvasc@gmail.com; 3 Department of Angiology and Vascular Surgery, Unidade Local de Saúde de Santo António, 4099-001 Porto, Portugal

**Keywords:** autotransplantation, nephrectomy, renovascular

## Abstract

**Background:** Nephrectomy with autotransplantation (NAT) has been performed as an alternative treatment for complex renovascular lesions, intricate ureteral strictures and nephron-sparing surgery in complex renal tumors. **Methods**: A retrospective observational study was conducted including patients who underwent a NAT from January 2010 to September 2023. Data collected included surgery indications, surgical technique, complications according to Clavien–Dindo classification and mean hospital stay. Descriptive and inferential statistical analysis was performed using IBM^®^ SPSS^®^ Statistics version 28.0.1.0. **Results:** A total of 34 consecutive patients underwent 38 NATs at our institution. Surgery indications were complex renovascular conditions in 35 cases (92.1%), of which 24 had renal artery aneurysms, and ureteral injuries in 3 cases (7.9%). Thirty-four kidneys (89.5%) were retrieved through a laparoscopic approach. No significant difference was observed between post- and pre-operative creatinine levels (0.81 vs. 0.72, *p* = 0.303). Early high-grade complications developed in 12 procedures (31.6%). Median cold ischemia time was significantly longer in patients who developed complications (163.0 vs. 115.0, *p* = 0.010). The median hospital stay was 10 days (8–13). The median follow-up was 51.5 months. **Conclusions:** NAT emerges as a successful therapeutic strategy for a highly select group of patients dealing with intricate ureteral lesions and kidney vascular abnormalities, demonstrating positive outcomes that endure in the long term.

## 1. Introduction

Nephrectomy with autotransplantation (NAT) was first described by Hardy in 1963 for the management of complex ureteral strictures [1]. Since then, NAT has been performed as an alternative treatment for extensive ureteral injuries, complex renal artery aneurysms, renovascular hypertension and nephron-sparing surgery in complex renal tumors and in chronic loin pain syndrome [2].

At present, the most common indication for NAT is complex and proximal ureteral strictures [3]. Urinary reconstruction for complex ureteral strictures poses a challenge, and the available long-term solutions are restricted [4]. The use of bowel interposition, often incorporating the terminal ileum, has demonstrated significant improvement, resulting in stable or improved renal function in 74.7% of patients [5]. Although successful in most, bowel substitution disrupts the gastrointestinal tract, leading to both short- and long-term complications and potentially being catastrophic [6]. Using the ileum as a urinary conduit can result in mucus plugging, stones, urinary tract infections, strictures, fistulas, small bowel obstructions and metabolic acidosis [7]. Naude introduced open ureteroplasty with buccal mucosa graft in 1999 as a method to address complex ureteral strictures not amenable to ureterostomy [8]. This technique was proposed as a potentially less morbid and technically demanding alternative compared to ileal ureter replacement and renal autotransplantation. Zhao et al., in 2015, first reported robotic ureteroplasty as a minimally invasive alternative method for proximal or multifocal ureteral strictures not amenable to primary anastomosis [9]. A multi-institutional retrospective review demonstrated that, with a median follow-up of 27.5 months, 87.0% of cases achieved surgical success. The current literature regarding ureteral reconstruction with buccal mucosa grafts is limited to a handful of small case series, thereby restricting the assessment of the safety and efficacy of this technique [10]. Trenti et al. described a novel approach to treat long complex strictures involving the proximal and the middle ureter using a free peritoneal graft that preserves any residual vascular supply of the ureter [11]. Following a longitudinal ureteral incision, a free peritoneal flap was obtained, secured as an onlay-patch onto the remaining ureteral plate and, finally, enveloped with the omentum. After a mean follow-up of 69.5 months, 63.6% of patients were free from recurrence without dilatation of the upper urinary tract and normal renal function, and only one patient required further surgery.

Although vascular anomalies are still a major indication for NAT, this method has been used less frequently, possibly because minimally invasive approaches are preferred [12]. However, some conditions are not suitable for interventional or endovascular therapies or in situ surgical reconstruction because of anatomical characteristics [13]. Ex vivo repair is recommended for complex aneurysms involving multiple branches and distal aneurysms, particularly when the anticipated renal ischemic time is likely to exceed 30–60 min [14]. Van Rooden et al. described ex vivo reconstruction and autotransplantation in patients with spontaneous renal artery dissection, primarily associated with fibromuscular dysplasia and presenting as arterial hypertension and renal dysfunction [15]. The use of NAT in the treatment of nutcracker syndrome (NCS) has been reported with good results, even in cases where left renal vein transposition or venous stenting has failed [16].

Laparoscopic nephrectomy may decrease the morbidity associated with autotransplantation [17].

In this study, we report the indications, surgical technique, complications and long-term outcomes of NAT in a 13-year period at our institution.

## 2. Materials and Methods

A retrospective observational study was conducted including patients who underwent NAT from January 2010 to September 2023. Patient demographic and baseline characteristics, including age, sex and comorbidities, were obtained. Surgery indications and clinical manifestations during the pre-operative evaluation were also extracted. Peri-operative data including surgical approach (open/laparoscopic), surgical time, ischemia time and complications according to Clavien–Dindo classification were described. Post-operative surgical complications were recorded as well as the duration of hospital stay. Hypertension was also evaluated and classified into three groups: (a) cured (patients achieved normotension and took no antihypertensive drugs); (b) improved hypertension (the number and dose of antihypertensive drugs were decreased after NAT); and (c) not-cured hypertension (patients still experienced high blood pressure and antihypertensive drugs were continued as before). Post-operative mortality was defined as all death events occurring within 30 days of the procedure. Long-term follow-up data on autograft durability and late complications were also extracted.

All patients underwent NAT by the same multidisciplinary team of urologists and vascular surgeons. Concerning the surgical technique, it was generally performed in accordance with the principles of living-donor kidney transplantation. Open nephrectomy was performed through lumbotomy, with proximal dissection of the vessels and retrieving the ureter with the largest length possible. Laparoscopic nephrectomy was carried out using a 4-port transperitoneal access, securing the pedicle with vascular clips. Once retrieved, the kidney was perfused with Celsior solution. When vascular reconstruction was needed, the great saphenous vein, superficial femoral vein, epigastric vein or ovarian vein were used. In patients with renal artery aneurisms, the basis of treatment involves excising the aneurysmal sac and then restoring in-line circulation to as many branches as possible. Ex vivo repairs of aneurysms include aneurysmorrhaphy, aneurysmectomy plus arteriorrhaphy, aneurismectomy associated with patch angioplasty, aneurysmectomy with bypass, clipping and clipping plus arteriorraphy. Vascular anastomoses to reperfuse the kidney were performed in an end-to-side fashion to external iliac vessels. Finally, ureteroneocystostomy was performed with the direct ureterovesical implantation Paquin technique, which involves forming an intravesical nipple from the distal ureter and ureteral stent placement. A Foley catheter was inserted into the bladder for 5 days. A suction drain was placed routinely and removed within 48 h or when the drain volume was <50 mL. Follow-up imaging was performed using ultrasonography with color Doppler on the first post-operative day. The ureteral stent was removed by flexible cystoscopy 4–6 weeks after NAT.

Statistical analysis was performed using the IBM^®^ SPSS^®^ Statistics version 28.0.1.0 software. Results for continuous variables were expressed as mean ± standard deviation or as median (interquartile range) according to their distribution. Categorical variables were described as absolute and relative frequencies. The chi-square test and Fisher’s Exact Test were applied to compare categorical variables. The independent sample Student *t*-test or the Mann–Whitney U test were used to compare continuous variables. The level of statistical significance adopted was *p* < 0.05.

## 3. Results

### 3.1. Demographic and Pre-Operative Data

A total of 34 consecutive patients underwent 38 NATs at our institution, including 4 patients who underwent staged bilateral NAT. Twenty-three (67.6%) patients were women. The patients’ median age was 48 years (38–61). Surgery indications were complex renovascular conditions in 35 cases (92.1%), of which 24 had renal artery aneurysms and 6 renovascular hypertension, and ureteral injuries in 3 cases (7.9%). Demographic and clinical characteristics are presented in Table 1. 

Hypertension was the most common comorbidity, affecting 22 patients (64.7%).

Three patients had complex bilateral aneurysms. In most patients with unilateral aneurysms, the right renal artery was affected (66.7%). Regarding the location of aneurysms, 16 (66.7%) were at the bifurcation and 4 at the bifurcation and in the first branch of its division. The median diameter was 2.3 (1.8–3.0). Multiple aneurysms were present in six cases (25.0%).

Of patients with renovascular hypertension, three had documented fibromuscular dysplasia. One of them had a history of renal artery rupture requiring emergency nephrectomy.

Of patients diagnosed with NCS, all had documented gross hematuria and one flank pain.

The causes of ureteral injuries were complete avulsion during ureteroscopy (URS), failed ureteropelvic junction repair and long ureteral stricture related to Crohn’s disease.

### 3.2. Peri-Operative Data

Thirty-four kidneys (89.5%) were retrieved through a laparoscopic approach.

The mean surgical time was 324 min (±70) and there was no statistically significant difference between the laparoscopic and open nephrectomies before NAT (300 ± 64 vs. 327 ± 71; *p* = 0.452). The mean nephrectomy time was 104 min (±43). The median warm ischemia time was 143 s (165–300) and the median cold ischemia time was 138 min (102–173). Table 2 lists surgical details. 

Renal vein elongation repair was required in 21 of the procedures (55.3%). The venous reconstruction was made with a spiral saphenous veins prothesis, a superficial femoral vein or an ovarian vein. Regarding ex vivo aneurysm reconstruction, the most performed procedures were aneurysmectomy associated with patch angioplasty (23.7%) and aneurysmectomy plus arteriorrhaphy (18.4%).

There were intra-operative complications in five procedures (13.2%)—stenosis of vascular anastomosis requiring reconstruction and vascular thrombosis.

### 3.3. Post-Operative Data

No significant difference was observed between post- and pre-operative creatinine levels (0.81 vs. 0.72, *p* = 0.303). None of the patients required hemodialysis.

Early high-grade complications (grade IIIa or greater) developed in 12 procedures (31.6%)—8 vascular thromboses requiring nephrectomy, 2 perirenal hematomas requiring surgery and 2 surgical site infections requiring reintervention. The most common complications were infections and hematuria. A higher mean surgical time was associated with post-operative complications of any grade (297.9 vs. 344.3 min, *p* = 0.0.37). The median cold ischemia time was longer in patients developing post-operative complications (163.0 vs. 115.0 min, *p* = 0.010). Post-operative data and complications are shown in Table 3. 

The median hospital stay was 10 days (8–13).

The median follow-up was 51.5 months. No additional graft losses were identified by post-operative imaging or by creatinine as a surrogate marker for graft survival.

Hypertension was cured in 6 (27.3%), improved in 5 (22.7%) and unresolved in 11 patients (50.0%).

One patient succumbed to metastatic sarcoma of the autotransplanted kidney.

## 4. Discussion

NAT is generally reserved for critical situations, and it frequently stands as the ultimate alternative prior to considering nephrectomy. It was first described by Hardy in 1963 for the treatment of complex ureteral strictures [1]. A few years later, this technique also gained importance in the treatment of complex renovascular anomalies, including renal artery stenosis and aneurysms [18,19]. Mainly due to successful endovascular interventions, many of these anatomical variants can now be managed through minimally invasive approaches, thereby limiting the usefulness of NAT. However, when minimally invasive techniques have failed or are not feasible, NAT remains a viable option with acceptable outcomes [20,21]. In our series, vascular anomalies comprised 92.1% of the indications for NAT. Endovascular procedures are associated with specific complications such as renal artery dissection, partial renal infarction, post-embolization syndrome, stent stenosis and stent or coil migration [22]. While less frequently documented, some of these complications may require further interventions. In situ repair requires renal artery clamping and is a viable choice when the predicted renal warm ischemia time is less than 30–60 min. In complex aneurysms that include distal or multiple renal arteries, ex vivo repair with autotransplantation may be a safer approach, allowing for adequate exposure and cold perfusion of the kidney during the repair process, ultimately minimizing the warm ischemia time and protecting renal function [14]. This approach has demonstrated both safety and effectiveness, with post-operative mortality rates varying between 0 and 9.6% [23,24,25]. Chandra et al. compared in situ and ex vivo reconstructions and observed no significant differences in morbidity, mortality or reoperation rates [26]. Regarding renal artery stenosis associated with various causes such as fibromuscular dysplasia, significant reductions in blood pressure and the number of anti-hypertensive drugs have been documented in most patients after NAT. These outcomes in patients with renal artery aneurysms exhibit more variability [16]. In our series, hypertension was cured in 6 (27.3%), improved in 5 (22.7%), and unresolved in 11 patients (50.0%). NCS describes the clinical symptoms associated with anatomical compression of the left renal vein. Renal autotransplantation, through the repositioning of the kidney, eliminates the compression point and may even address kidney ptosis if present. Bath et al. documented three patients treated for loin pain hematuria syndrome attributed to NCS after unsuccessful left renal vein transposition, relapsing with debilitating pain. Subsequently, two of these patients underwent NAT, leading to the complete resolution of symptoms [27].

Ureteral stricture is considered the main indication for NAT, accounting for 70–80% of the cases reported in the literature during the last decade [3,28]. In our experience, this was the second most common indication for NAT. One alternative to NAT is ileal interposition. We believe that NAT holds an advantage over ileal interposition by preserving the ureter as the conduit, thereby avoiding the potential risks derived from bowel interposition, including urinary tract infections, acidosis, mucus production and intestinal complications [5,29]. Recently, ureteral reconstruction with buccal mucosa and free peritoneal grafts was highlighted as an effective treatment option for the management of complex proximal and middle ureteral strictures, with low morbidity and excellent intermediate-term outcomes. Nonetheless, further studies evaluating risk factors for failure are imperative to refine patient selection for these techniques [1]. The absence of a comparative analysis between the different approaches poses a challenge in assessing the true effectiveness and safety of the procedures.

Patients with large and complex hilar renal cell carcinoma, particularly in solitary kidneys, or patients with chronic kidney disease have also undergone treatment with NAT after ex vivo partial nephrectomy and bench surgery. However, due to unsatisfactory oncological outcomes, marked by 25–50% relapse, and functional outcomes, with 14–21% decreased graft function, we have not used this technique in the setting of RCC [3,19,30,31].

At present, a trend exists in favor of laparoscopic nephrectomy. While long-term outcomes and major complications remain comparable between laparoscopic and open nephrectomy, opting for the minimally invasive approach offers several advantages, including a reduced risk of surgical site infections and a shorter length of hospital stay [32]. Tran et al. [3] documented the most extensive laparoscopic series to date, encompassing a total of 52 patients, of which 90.3% exhibited long-term kidney functionality after a follow-up period of 73.5 months. Cowan et al. [33] conducted the first multi-institutional study, with a total of 54 procedures, with 76.5% involving open kidney retrieval and 23.5% employing the laparoscopic approach. Despite a shorter follow-up duration, the functional outcomes were comparable, with a 93% rate of functioning grafts.

Renal vein elongation repair was required in 21 of the surgeries (55.3%), mostly in the right kidney (90.5%). Nevertheless, it should be employed only when necessary. Choosing the appropriate material for vascular reconstruction constitutes a crucial decision [34]. Consistent with most series [23,35], our approach favors the use of the saphenous vein as the primary conduit for both arterial and venous reconstruction, followed by the superficial femoral vein for venous reconstruction. Notably, the ovarian vein has been abandoned from vascular reconstruction due to its inherent fragility and immediate rupture in some patients undergoing living donor transplant [34].

Cold ischemia time should be as short as possible since it has been described as a predictor of post-operative complications [33]. In our study, a longer cold ischemia time was associated with post-operative complications (163.0 vs. 115.0 min, *p* = 0.010). The two primary factors contributing to prolonged ischemia time include the requirement for meticulous bench surgery in renovascular cases and the necessity to conclude the nephrectomy procedure before initiating the autotransplantation.

After a 51.5-month follow-up, 79% of the patients have a functioning graft. All kidney losses occurred in the early post-operative period due to vascular thrombosis. These findings align with the existing literature, where graft survival typically ranges from 60% to 100%. It is crucial to acknowledge that vascular thrombosis is a complex interplay of surgical, vascular and patient-specific factors. The occurrence of this complication can be linked to laborious vascular reconstruction, leading to prolonged cold ischemia time and unpredictable anastomotic complications. Minimizing these risks requires careful surgical technique, thorough pre-operative evaluation, continuous post-operative monitoring and prompt intervention in the case of complications.

This study has some limitations, such as its retrospective nature and small sample size. Post-operative imaging was conducted at the discretion of the surgeons. Consequently, serum creatinine was often utilized as a surrogate marker for transplant survival.

## 5. Conclusions

NAT proves to be an effective therapeutic approach for a highly select group of patients with kidney vascular abnormalities, demonstrating favorable outcomes even in the long term. Regarding ureteral strictures, this procedure should be reserved for situations where all previous repair attempts have proven unsuccessful, and NAT remains the final resource to preserve the organ.

The success of NAT is intricately linked to the synergy of a collaborative multidisciplinary team with extensive experience in living-donor kidney transplantation.

## Figures and Tables

**Table 1:** Demographic and clinical characteristics.

Female—*n* (%)	23 (67.6)
Age at surgery, years—median (IQR)	48 (38–61)
Hypertension (yes)—*n* (%)	22 (64.7)
BMI, kg/m^2^—mean (SD)	25.6 (4.8)
Pre-operative creatinine level, mg/dL—median (IQR)	0.72 (0.63–0.89)
Surgical indications—*n* (%)	
*Complex renovascular conditions*	35 (92.1)
Renal artery aneurysms	24 (63.2)
Renovascular hypertension	6 (15.8)
Nutcracker syndrome	4 (10.5)
Type 3c endoleak following endovascular abdominal aortic aneurysm repair.	1 (2.6)
*Ureteral injuries*	3 (7.9)
Complete ureteral avulsion	1 (2.6)
Failed ureteropelvic junction repair	1 (2.6)
Long ureteral stricture (Crohn’s disease)	1 (2.6)

**Table 2:** Surgical details.

Surgical time—mean (SD)	324 (70)
Nephrectomy—*n* (%)	
Open	4 (10.5)
Laparoscopic	34 (89.5)
Nephrectomy time—mean (SD)	104 (43)
Ischemia time—median (IQR)	
Warm, seconds	143 (165–300)
Cold, minutes	138 (102–173)

**Table 3:** Post-operative data and complications.

Length of stay, days—mean (SD)	10 (8–13)
Short-term complications (Clavien–Dindo)—*n* (%)	
Low-grade (I-II)	10 (26.3)
High-grade (IIIa-V)	12 (31.6)
Post-operative creatinine level, mg/dL (at discharge)—median (IQR)	0.81 (0.61–1.0)
Hypertension—*n* (%)	
Cured	6 (27.3)
Improved	5 (22.7)
Unresolved	11 (50)

## Data Availability

No new data were created or analyzed in this study.
